# Use of 3D printers to create a patient‐specific 3D bolus for external beam therapy

**DOI:** 10.1120/jacmp.v16i3.5247

**Published:** 2015-05-08

**Authors:** Sarah Burleson, Jamie Baker, An Ting Hsia, Zhigang Xu

**Affiliations:** ^1^ Department of Radiation Oncology Stony Brook Medicine Stony Brook New York USA

**Keywords:** bolus, 3D printer, compensator, electron conformal therapy

## Abstract

The purpose of this paper is to demonstrate that an inexpensive 3D printer can be used to manufacture patient‐specific bolus for external beam therapy, and to show we can accurately model this printed bolus in our treatment planning system for accurate treatment delivery. Percent depth‐dose measurements and tissue maximum ratios were used to determine the characteristics of the printing materials, acrylonitrile butadiene styrene and polylactic acid, as bolus material with physical density of 1.04 and $1.2\;g/{\text{cm}}^{3}$, and electron density of $3.38\times 10^{23}\text{electrons}/{\text{cm}}^{3}$ and $3.80\times 10^{23}\;\text{electrons}/{\text{cm}}^{3}$, respectively. Dose plane comparisons using Gafchromic EBT2 film and the RANDO phantom were used to verify accurate treatment planning. We accurately modeled a printing material in Eclipse treatment planning system, assigning it a Hounsfield unit of 260. We were also able to verify accurate treatment planning using gamma analysis for dose plane comparisons. With gamma criteria of 5% dose difference and 2 mm DTA, we were able to have 86.5% points passing, and with gamma criteria of 5% dose difference and 3 mm DTA, we were able to have 95% points passing. We were able to create a patient‐specific bolus using an inexpensive 3D printer and model it in our treatment planning system for accurate treatment delivery.

PACS numbers: 87.53.Jw, 87.53.Kn, 87.56.ng

## I. INTRODUCTION

Bolus is very useful for the treatment of shallow tumors, increasing skin dose, and improving dose uniformity. Patient‐specific bolus is designed for shaping the desired dose surface to conform and contain the planning target volume (PTV) while delivering minimal dose to adjacent, underlying critical structures and normal tissues. Currently, the use of a solid machinable bolus device placed directly on the patient's skin is gaining popularity. Several clinical studies have shown the clinical efficacy of this technique for head and neck,^1^, ^2^ post mastectomy irradiations,^3^, ^4^ and paraspinal muscle treatment.^5^, ^6^ Compared to Bolus Electron Conformal Therapy (BolusECT), which uses milling machines, 3D printers have less operational and production costs. Using 3D printers for radiation therapy has been gaining interest;^7^, ^8^, ^9^ however, not much is known about how therapeutic radiation beams interact with the materials used in 3D printers and how they compare to other bolus materials. In this project, we explain the simple process of designing and printing a bolus using our treatment planning system and an inexpensive, less than $3,000, 3D printer (Airwolf 3D, Costa Mesa, CA). A comprehensive study is done measuring the properties of two printing materials used with our printer, allowing us to model these materials within our treatment planning system. We investigate further by printing a patient‐specific 3D bolus for the Alderman RANDO phantom and create a treatment plan for the RANDO phantom and the custom 3D bolus, simulating a real patient treatment. The calculated dose plane from that plan is compared to one measured using film in order to compare the efficacy of the printed bolus.

## II. MATERIALS AND METHODS

### A. Printing of 3D bolus

**Figure 1:**
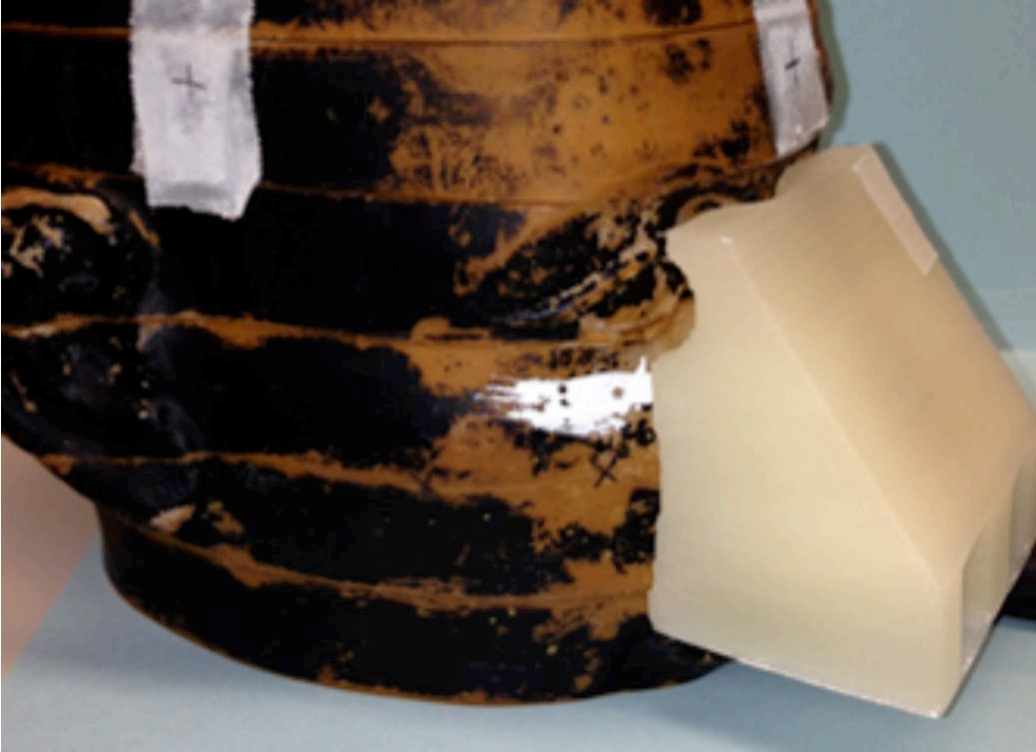
Example of bolus printed to conform to the nose of the Alderson RANDO phantom

The design process of the 3D bolus requires a CT scan of the patient. For this study we used the Alderson RANDO phantom (The Phantom Laboratory, Salem, NY) as our test subject. After the CT study is acquired, the data was exported to Varian Eclipse (Varian Medical Systems, Inc, Palo Alto, CA) treatment planning system. A customized bolus was added as a structure and the shape can be optimized to meet prescription and coverage requirements. As seen in Fig. 1 the bolus structure can be designed to any shape, limited only by the printer. Dose calculation by Varian Eclipse Electron Monte Carlo (eMC) was used for the project. The eMC has five user‐selectable parameters for individual calculations: calculation grid size, accuracy, number of particle histories, smoothing method, and smoothing level. To attain accurate calculations, a dose grid of 1 mm 1% error accuracy level determined the number of particles, 3D Gaussian smoothing method and a smoothing level of medium was used in the dose calculation.

**Figure 2:**
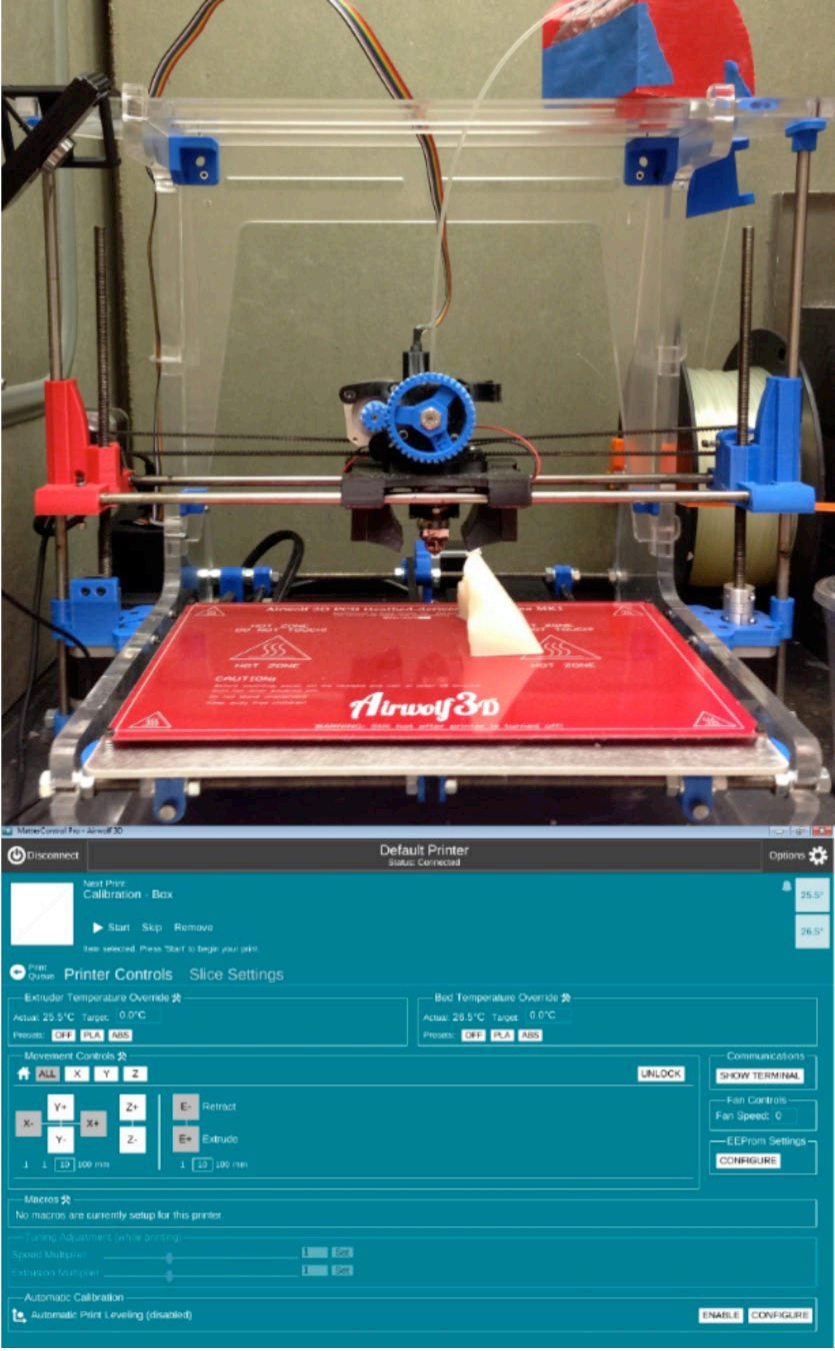
The Airwolf printer model, XL 3D, is seen above, and the MatterControl Pro software interface can be seen below that. A spool of PLA material is connected to the printer extruder head. An already printed object is shown sitting on the printing bed. During a print, the extruder head would be heating up the PLA filament and depositing, layer by layer, the design exported by the software. The software also controls desired printer and slice settings seen in the screen shot below.

Once a satisfactory bolus design was determined, the structure set was exported to 3DSlicer (www.slicer.org), which is a 3D modeling software that is maintained as open source with many developers. The SlicerRT extension must be installed to support dicom structures and doses. 3DSlicer exports the bolus in standard tessellation language (STL), which is a file format that describes 3D volumes using triangles with adjoining edges. The STL files were interpreted by the 3D printer software MatterControl Pro (Airwolf 3D). MatterControl Pro then sends instructions to our XL 3D printer (Airwolf 3D). The MatterControl Pro software and the printer we used can be seen in Fig. 2.

The shapes and sizes of bolus that are printable are greatly dependent on the 3D printer used. The printer we used, the Airwolf XL 3D printer, can print bolus with maximum dimensions of 30 cm x 20 cm x 18 cm. In addition, the XL 3D printer only prints in one material at a time, so we were limited in the bolus designs that are supported from the bottom. This restricts printing of overhangs to an angle of about 45° from horizontal depending on the printer settings. A printer that uses two materials at once can support the bolus material by printing a support structure with polyvinyl alcohol (PVA) while printing the intended bolus structure on top with another material. This enables any bolus design to be printed. PVA is water‐soluble, so it is easily removed from the bolus material by rinsing the bolus in water. Other important factors to consider while printing are the settings used while printing because they can alter the density of the printed bolus. Ideally, the 3D printer creates a solid bolus material with no air gaps. However, a truly solid bolus is currently unachievable. Adjusting the print settings so that this is best achieved is important and can be different depending on the 3D printer model and setup. For example, different fill patterns and fill densities can be chosen. It is important to note that each combination of these settings that will be used should be investigated separately by each facility in order to accurately determine the physical and electron densities of a printed bolus. The printer settings that we used for the material polylactic acid or PLA can be seen in Table 1.

**Table 1:** Airwolf 3D printer settings.

*Print Settings*	
Bed Temperature	70°C
Extruder Temperature	190°C
Layer Height	0.3 mm
Extrusion Width	1 mm
Layer Speed	25 mm/s
Perimeter Speed	17 mm/s
Fill Density	1
Fill Pattern	rectilinear
Fill Angle	45°
Perimeter Extrusion Width	0.5 mm
Nozzle Diameter	0.5 mm
Filament Diameter	2.78 mm
Extrusion Multiplier	1
Z Offset	0.1
*First Layer Settings*	
Bed Temperature	100°C
Extruder Temperature	200°C
Layer Height	0.5 mm
Extrusion Width	1.5 mm
Layer Speed	12.5 mm/s

Ensuring that the bolus remains attached to the print bed can be difficult, depending on the shape being printed. We found that the best results to combat this were achieved when Elmer's Glue‐All is diluted with water to a concentration of 20% and spread on the print bed. This mixture is allowed to dry before printing the bolus on top of it. After printing, the bolus is easily removed from the print bed by rinsing with water.

### B. Plastics used for 3D printed bolus

Two plastic materials were tested for this project: a red acrylonitrile butadiene styrene (red‐ABS) and a clear polylactic acid (clear‐PLA). The physical properties for the materials and water are listed in Table 2.

**Table 2:** Physical properties of red‐ABS, clear‐PLA, and water.

*Physical Property*	*Red‐ABS*	*Clear‐PLA*	*Water*
Chemical Formula	C_8_H_8_•C_4_H•C_3_H_3_N	C_3_H_4_O_2_	H_2_O
Hydrogen Content (by mass)	8%	6%	11.1%
Physical Density $\left(g/{\text{cm}}^{3}\right)$	1.04	1.2	1
Electron Density ratio compared to water	1.01	1.14	1
Effective Z	3.45	4.22	3.33

Ideally bolus material should be tissue‐equivalent, having similar properties to water. The red‐ABS plastic more closely resembles the properties of water, having almost the same electron density, effective Z, and physical density. In radiation therapy energies, Compton interactions dominate in any medium for photon beams, while electrons are primarily interacting with the electric fields of other charged particles.^(10)^ Therefore, restricted stopping power and consequently absorbed dose is greatly affected by mass density and electron density.^5^, ^11^ These physical properties, as well as ionization measurements, will be used to choose a HU value for the bolus created in Eclipse. The correct assignment of HU value is crucial for treatment planning purposes to use these materials in the clinic as bolus.

### C. Tissue maximum ratio (TMR) and percent depth‐dose (PDD) measurements

TMR measurements were done using a plane‐parallel ion chamber set at the isocenter (Markus chamber, active volume 5.5 cc) (PTW, Hicksville, NY) with different thicknesses of the printed plastic slabs placed on top. Red‐ABS and clear‐PLA with thicknesses of 2–40 mm were tested. Two hundred (200) MUs of 6 MV photons were used for the TMR measurements with a $4\times 4\;{\text{cm}}^{2}$ field. The two plastic materials were compared to the same TMR measurements in water and to the same TMR measurements of wax bolus and Superflab bolus materials (Mick Radio‐Nuclear Instruments Inc., Mount Vernon, NY). Thicknesses of 5 mm to 30 mm of wax and Superflab bolus materials were used in this study to replicate current clinical use.

PDD measurements were also done using the Markus chamber. A photon beam of 6 MV and electron beams of 9 MeV and 12 MeV were used for the measurements. For the photon beam, a field size of $4\times 4\;{\text{cm}}^{2}$ was used and for the electron beam, a $6\times 6\;{\text{cm}}^{2}$ cone with a $4\times 4\;{\text{cm}}^{2}$ cutout was used. Measurements were completed by placing the pieces of plastic on top of the ion chamber with a source‐to‐surface distance (SSD) of 100 cm to the phantom surface. All measurements were performed on a Varian Silhouette Edition Clinac Sil21IX (Varian Medical Systems) linear accelerator. The ionization measurements were converted to dose using the ratio of the collisional stopping powers of the bolus material to air. The collisional stopping powers for each material at various energies were obtained from the NIST program ESTAR.^(12)^ The PDD results from the bolus material were compared to PDD measurements in water and to various materials using calculated data from our treatment planning system.

### D. Dose plane comparison

In this experiment we used a printed custom bolus and exposed Gafchromic EBT2 film (lot # 07301303) (International Specialty Products, Wayne, NJ) to obtain planar dose distributions in the Alderson RANDO phantom using a 9 MeV electron beam. The planar dose distributions collected with the film were then compared to dose planes calculated using our treatment planning system. The custom bolus was designed to treat the RANDO phantom nose using the method described in the Materials & Methods section A. All radiation exposures were performed on a Varian Silhouette Edition Clinac Sil21IX (Varian Medical Systems) linear accelerator.

The EBT2 film measurements were analyzed using MapCHECK film QA software (Sun Nuclear, Melbourne, FL). An optical density versus dose calibration curve was obtained for the film batch used in the study. The film was calibrated using 9 MeV electrons, but is valid for any energy used in the study because of the minimal energy dependence.^(13)^ Prior to film irradiation, the output of the linear accelerator was confirmed according to TG‐51. Films were placed at a depth of 2 cm for 9 MeV electron beam, in slabs of solid water with sufficient solid water below the film for adequate back scatter. A $10\times 10\;{\text{cm}}^{2}$ electron cone with standard insert was used to deliver accurate doses ranging from 0.1 Gy–3 Gy in six increments. The irradiated EBT2 films were scanned into the MapCHECK software with an Epson 10000XL (Seiko Epson Corp, Nagano, Japan) flatbed color scanner, in a method similar to that described by Andrés et al.^(14)^

Planar dose distributions were measured by placing the EBT2 films between slabs of the RANDO phantom head at the bottom of the nose. The custom 3D printed bolus that was created using the CT of the RANDO phantom was placed on top of the RANDO nose. Then using 9 MeV electrons with a $10\times 10\;{\text{cm}}^{2}$ cone and $4\times 4\;{\text{cm}}^{2}$ cutout, at 105 cm SSD, the printed bolus and RANDO phantom were irradiated with 200 MU at a 34° angle to the plane of the EBT2 film. The MapCHECK software was designed to correctly analyze EBT2 film using the calibration curve created earlier. After importing the scanned images into the MapCHECK software, dose planes from the treatment planning system calculations were registered to the film and compared.

To provide comparisons of multidimensional dose distributions, dose comparison tools such as gamma dose distribution, distance to agreement (DTA), and dose difference (DD) were used.^(15)^ MapCHECK includes a selection of evaluation tools that can be used to compare the dose distribution of the films to the dose distribution of the calculations. The gamma map is a qualitative map that is a mathematical combination of the dose difference and difference‐to‐agreement calculations. The gamma dose distribution tool was used in our film analysis. We compared point measurements throughout the dose plane. We analyzed various gamma pixel parameters and decided to focus on the pixel passing rate with criteria of 5% dose difference, to reflect the inherent error in EBT2 film and 2 mm distance to agreement (DTA), ensuring that the larger dose gradient of the electron beams dose not artificially raise the gamma index.^(16)^

### E. OSLD study using nanoDot

**Figure 3:**
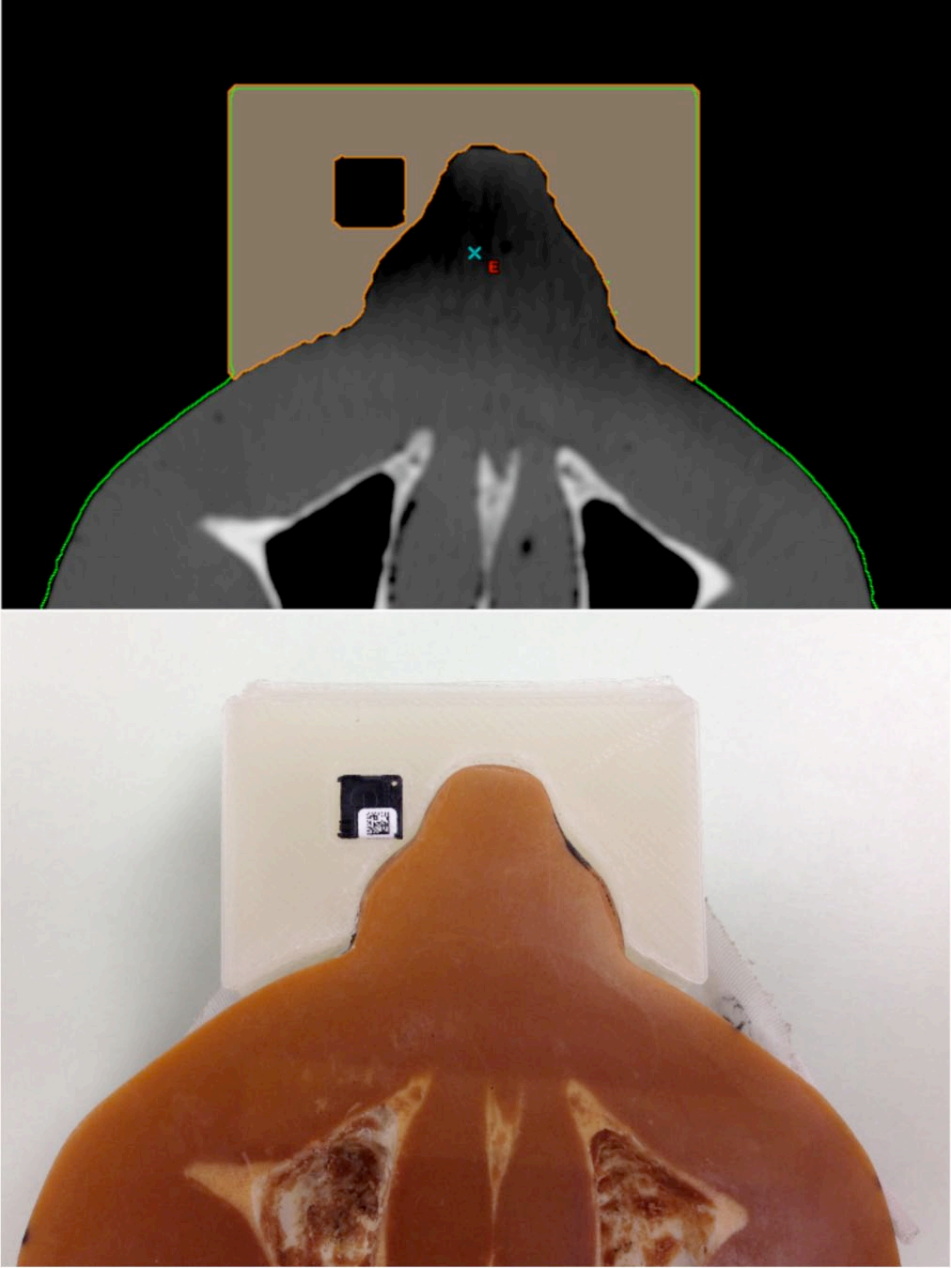
NanoDot placement within bolus for measurement. Digitial rendering from Eclipse seen on top and actual photo seen below.

For further dosimetric validation of our printed bolus, we conducted a study using nanoDots (Landauer, Glenwood, IL). A new bolus was printed out with a compartment for the nanoDot, shown in Fig. 3. The RANDO phantom and the new bolus with the nanoDot was irradiated with 9 MeV electrons using similar methods described in the Materials & Methods section D. The nanoDots were read and compared to treatment planning calculations.

## III. RESULTS & DISCUSSION

### A. TMR measurements using 6 MV photon

**Figure 4:**
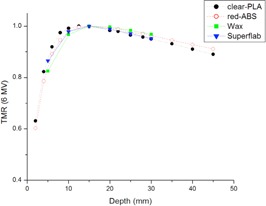
TMR curves using 6 MV photons of clear‐PLA and red‐ABS compared to the wax and Superflab bolus already used in the clinic. Clear‐PLA has a quicker buildup and then falls off at the same rate as the Superflab bolus. All other materials have a similar buildup region, with red‐ABS following the declining slope of the wax TMR curve.

In Fig. 4, TMR curves for the printing materials, red‐ABS, and clear‐PLA are compared to two bolus materials currently used in our clinic — wax and Superflab.

The difference between the TMR curves for the four materials is less than 4%. These data indicate that similar results will be achieved using either of the two printing plastics for bolus during treatment compared to the traditional bolus currently used.

**Figure 5:**
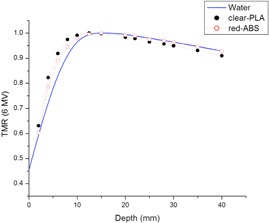
TMR curves of water, red‐ABS, and clear‐PLA. All measurements were normalized to $D_{\text{max}}$. Although both plastics have a similar curve compared to water, the red‐ABS follows the trend of water more closely.

In order to test the tissue equivalency of the two materials, we compare the measured 6 MV photon TMR curves in red‐ABS and clear‐PLA to measured TMR curves in water. This is seen in Fig. 5.

Red‐ABS is found to show a maximum deviation of 6% at a depth of 5 mm and a 0.12% deviation at a depth of 40 mm in ionization readings, compared with water. Clear‐PLA is found to show a maximum deviation of 9% at a depth of 5 mm and a 2.06% deviation at a depth of 40 mm in ionization readings, compared to water. Both materials differ from water in their buildup region, but red‐ABS follows a similar trend to water after $D_{\text{max}}$. However, after printing several objects with the red‐ABS, the integrity of the structure became compromised at a certain height, with layers separating or edges curving. Therefore, red‐ABS was not considered to be useful in the clinic with our current printer. Only the clear‐PLA was tested for the rest of the study.

### B. PDD measurements

Percent depth‐dose measurements for the clear‐PLA plastic were measured using a 6 MV photon beam and both 9 MeV and 12 MeV electron beams. These PDD curves were compared to those measured in water.

**Figure 6:**
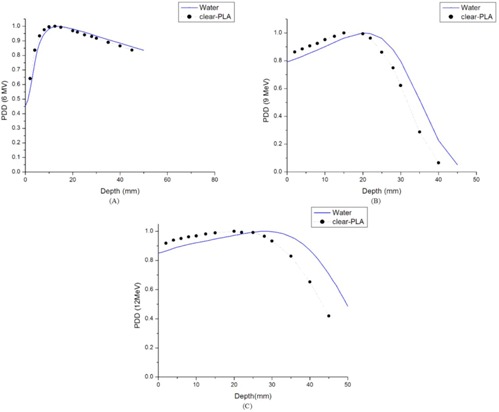
PDD curves comparing water and clear‐PLA: (a) the PDD curve comparing clear‐PLA and water for 6 MV photons with 4 cm×4 cm field size; (b) the PDD curve comparing clear‐PLA and water for 9 MeV electrons with a 4 cm×4 cm cutout size; (c) the PDD curve comparing clear‐PLA and water for 12 MeV electrons with a 4 cm×4 cm cutout size.

The PDD curve in clear‐PLA builds up quicker and falls off steeper than the PDD in water in both photons and electrons, as shown in Fig. 6. For 6 MV photons, shown in Fig. 6(a), $d_{\text{max}}$ in clear‐PLA is 1.5 mm shallower than water. 5, 6 show the same directional shift in $d_{\text{max}}$ between water and clear‐PLA for electrons. There is a shift in $d_{\text{max}}$ of 5 mm for 9 MeV electrons and a shift in $d_{\text{max}}$ of 8 mm for 12 MeV electrons, compared with water.

In the Material & Methods section A, we discussed the electron density and physical density for clear‐PLA and compared it to water. The clear‐PLA has a 14% higher electron density and 20% higher physical density than water. Our measured PDD curves for clear‐PLA compared to water reflect these physical differences between the materials. There is a greater build up and a steeper fall off after $d_{\text{max}}$ for clear‐PLA compared with water. Therefore, we cannot assume it is a water‐equivalent material and thus cannot use a Hounsfield unit (HU) number of 0 when doing our treatment planning calculations for clear‐PLA.

**Figure 7:**
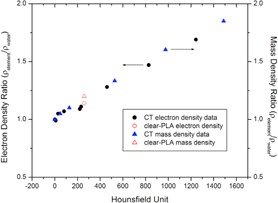
CT data from our clinic showing density ratios compared to water and the corresponding Hounsfield unit. A material with electron density ratio of 1.14 and mass density ratio of 1.2 corresponds to a HU of 260.

Using our clinical CT data (GE LightSpeed, GE Healthcare, Waukesha, WI), we found that a material with an electron density ratio compared to water around 1.14 and mass density ratio of 1.2 is equivalent to a material that has a HU number of 260. This is shown in Fig. 7.

**Figure 8:**
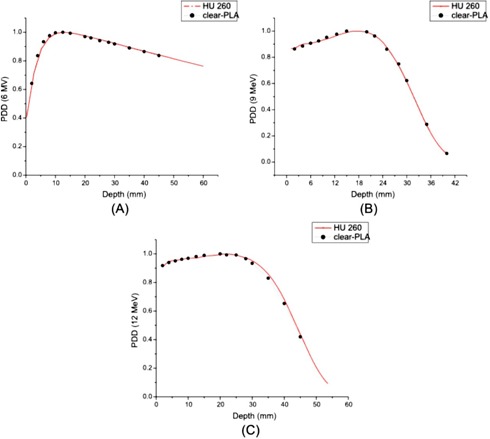
PDD curves comparing calculated data from Eclipse RTP for a material with a HU number of 260 and clear‐PLA: (a) the PDD curve comparing clear‐PLA and a material with a HU number of 260 for 6 MV photons with 4 cm×4 cm field size; (b) the PDD curve comparing clear‐PLA and a material with a HU number of 260 for 9 MeV electrons with a 4 cm×4 cm cutout size; (c) the PDD curve comparing clear‐PLA and a material with a HU number of 260 for 12 MeV electrons with a 4 cm×4 cm cutout size.

Therefore, in our treatment planning system we calculated the PDD for a material with a HU number of 260 and compared it to all the measured PDD curves in clear‐PLA, as shown in Fig. 8.

There is great agreement between the measured PDD curves in clear‐PLA for all beams and the calculated PDD curves from our treatment planning system using a HU number of 260. For 6 MV photons there is a 0.25 mm difference in the depth of $d_{\text{max}}$ and a 0.5% difference in PDD at 45 mm when comparing the calculated PDD data to the measured PDD data. A sixth degree polynomial curve is used to model the measured data for the 9 MeV and 12 MeV PDD due to the limited number of measurements around the $d_{\text{max}}$ point of the curve. For a 9 MeV electron beam, there is a less than 2% difference at a depth of 5 mm and less than 1% difference in PDD at 40 mm when comparing the calculated PDD data to the fitted PDD curve for the measured data. For a 12 MeV electron beam, there is a less than 1% difference at a depth of 5 mm and a 1% difference in dose at a depth of 45 mm between the calculated PDD data from and the fitted PDD curve for the measured data. These data strengthen our argument that we can correctly model clear‐PLA in our treatment planning system for accurate dose delivery.

The printing material was also imaged using our CT scanner and an average Hounsfield unit number of 160 was measured. This number was not consistent with the Hounsfield number results of our PDD measurements discussed previously. There are known discrepancies between electron density and Hounsfield unit number from CT scans depending on many factors like beam quality, technique used, position of the phantom, for example.^17^, ^18^ Considering this, the disagreement between the Hounsfield unit measured from PDD (260) and the Hounsfield number measured from the CT scanner (160) was considered justifiable. We feel that the more accurate representation of the printing material is the Hounsfield unit of 260, due to the more accurate methods of obtaining that number.

### C. Dose plane comparison results

**Figure 9:**
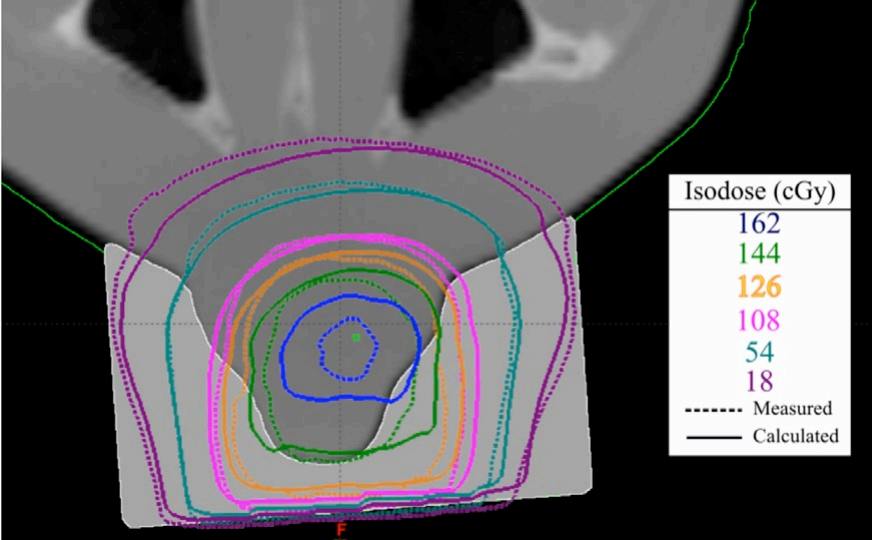
Dose plane comparison between a calculated dose plane from Eclipse RTP using a plan with graphically contoured bolus, and a measured dose plane using Gafchromic film. 86.5% of points were passing with gamma criteria of 2 mm DTA and 5% dose difference.

There is good agreement between the isodose distribution for 9 MeV with the 3D printed bolus at 105 cm SSD using $10\times 10\;{\text{cm}}^{2}$ cone and $4\times 4\;{\text{cm}}^{2}$ cutout. The MapCHECK data are shown in Fig. 9.

Various isodose lines are shown from 18 cGy–162 cGy. Doses under 10 cGy were ignored. The agreement between measured and calculated values was good with 86.5% of data points passing gamma requirements of 2 mm DTA and 5% dose difference.

**Figure 10:**
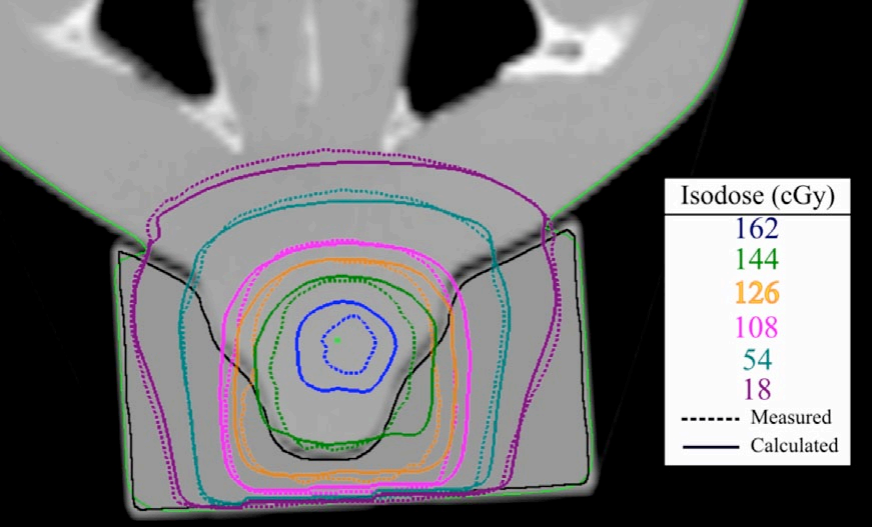
Dose plane comparison between the calculated dose plane from Eclipse RTP using a plan with a new CT with printed bolus, and the measured dose plane using Gafchromc film. 89.6% of points were passing with gamma criteria of 2 mm DTA and 5% dose difference.

To be able to make the bolus with little patient involvement and in minimal time, we propose that the patient does not need to have a second CT done with the printed bolus for treatment planning. In order to verify this, we did a second CT scan of the RANDO phantom with the printed bolus. The printed bolus density is overridden in Eclipse with a Hounsfield unit number of 260. Then we calculated the treatment plan on the new CT using the same delivery parameters as before and compared it to the film measurement taken. The result of that dose plane comparison is shown in Fig. 10.

With a second CT using the printed bolus on the patient, more points using the gamma criteria of 2 mm DTA and dose difference of 5% passed, going from 86.5% to 89.6%. The main difference in the second CT with the printed bolus are the slight air gaps between the bolus and the RANDO phantom that are accounted for in the calculation. Therefore, the calculated plan from that CT calculates a more accurate dose plane compared to what is actually delivered. However, we find that using either method is sufficient.

### D. OSLD study using nanoDot

In addition to our dose plane comparison study, we further investigated the accuracy of modeling the printed bolus in our treatment planning system by doing a dosimetric study using nanoDots. The calculated dose from the treatment planning system was 159.0 cGy and the average nanoDot measurement reading was 159.8 cGy. This corresponds to a −0.5% difference between calculated and measured dose, further validating our study.

### E. Quality assurance and clinical considerations

As with introducing any new equipment into a radiation oncology clinic, it is prudent to develop quality assurance procedures to ensure patient safety. The quality of the printed bolus product can be of concern for treatment planning. If a facility is choosing to not repeat a CT scan of a patient with the bolus, the integrity of the bolus can be assessed by simply scanning the bolus on its own through the CT. In that manner, the bolus can be analyzed for any holes, air gaps, structural damages or inconsistencies. The density of the bolus can also be relatively compared to previous bolus using the same material and technique.

To maintain sanitary conditions, plastic wrap is placed around the bolus before being placed on patients' skin. This ensures a sterile environment for every treatment. In addition to sanitary considerations, as with use of any bolus, skin dose to the patient during treatment will increase. Although this increase wasn't explicitly tested in this paper, one can see in Fig. 6, the PDD curves of the printed material compared with water, the $d_{\text{max}}$ of the bolus material is shifted toward the surface. This implies that the skin surface may receive more dose than traditional bolus (wax, Superflab). This should be considered during treatment by the physician and it is recommended the patient be closely monitored for severe skin erythema. It may be necessary to adjust the PLA bolus thickness to less than what is typically used to account for the extra skin dose.

Several practical considerations are discussed in order to implement the use of the 3D printer for bolus creation in a clinic. First, the typical printing time of a bolus, using our printer, was 4 to 6 hrs. The bolus placed on the RANDO phantom for dose plane comparisons took approximately 4 hrs to print. Larger objects, like some patient anatomy, can take an additional 1 to 2 hrs. Also, it is prudent to consider the failure of some bolus to print correctly on the first print, resulting in the need for extra time for final production. Next, possibility of radiation degradation of the printing material was considered. However after multiple irradiations (∼10−15) of our printed PDD phantom and our RANDO phantom nose bolus, no degradation was observed.

### F. Other applications: printing positive patient molds for traditional bolus fitting

In some cases traditional bolus such as wax, Superflab or Aquaplast RT (WFR/Aquaplast Corp., Avondale, PA) sheets are better suited for treating specific areas. However, if a patient has very sensitive skin or an open wound, fitting bolus to the patient's body can be painful and cause inaccuracies in placement. Instead we can print parts of patient's bodies and fit the bolus on our printed positive mold instead of on the patient. That way we can ensure accurate placement and a form fitting bolus without air gaps and without adding patient discomfort. We have been using this method to create bolus for patients getting scalp treatments. A typical sample for one patient being treated with 6 MV photons is shown in 11, 12.

**Figure 11:**
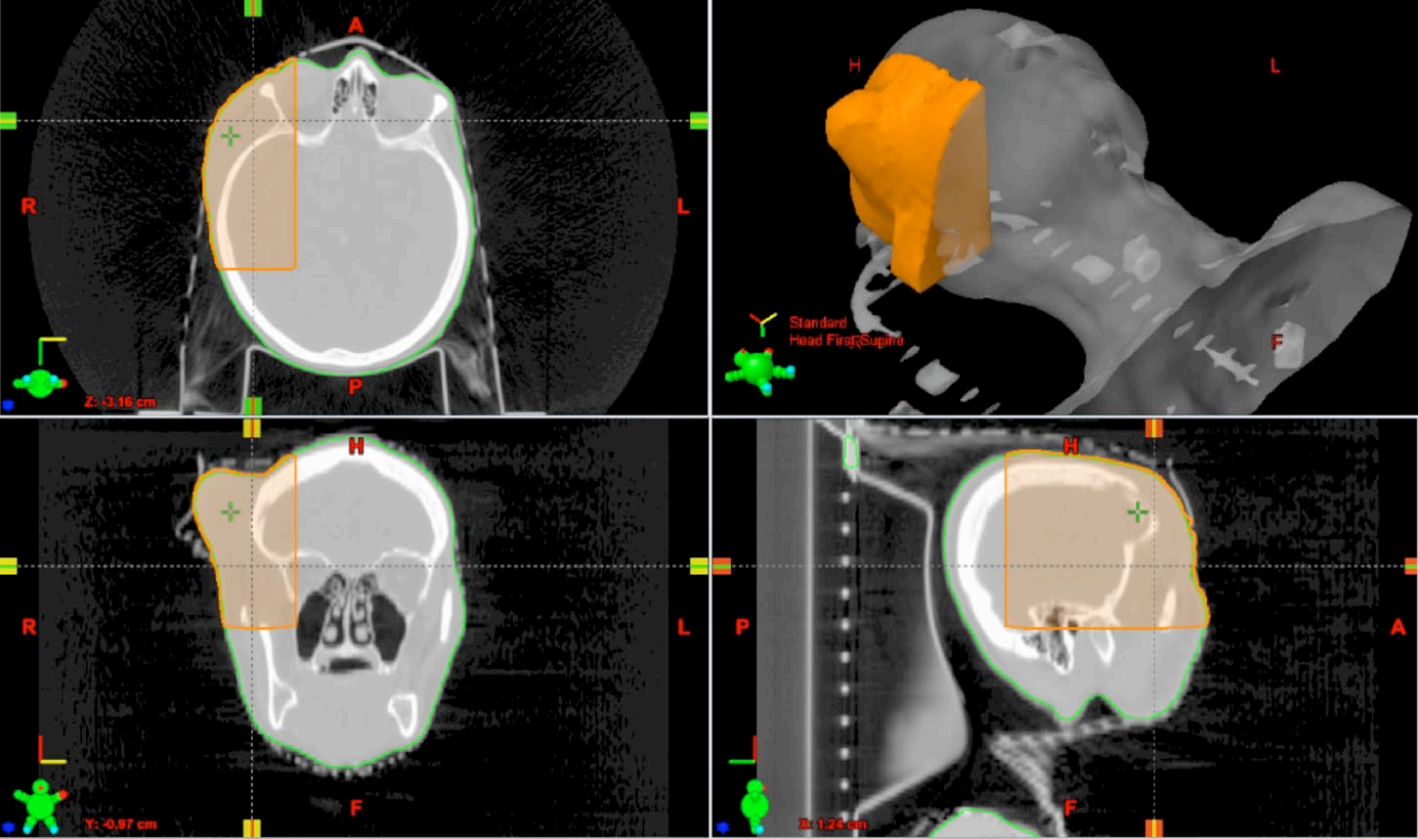
The process of designing a positive mold of a patient and printing for Aquaplast bolus fitting. Axial, coronal, and sagittal views from the CT of our patient are shown counterclockwise from the top left. The area highlighted in orange was the area sent to the 3D printer. A 3D rendering is seen in the top right corner.

**Figure 12:**
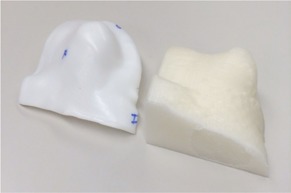
The printed 3D positive mold of the patient's scalp and molded Aquaplast RT sheet. The patient mold can be seen on the right, and the Aquaplast RT sheet that was heated and molded on top of the printed product can be seen on the left.

The anatomy sent to the 3D printer, highlighted in orange, is shown in Fig. 10. This is the area of the patient where the Aquaplast RT bolus was to be placed. This area on the patient was too sensitive for the heated Aquaplast RT to be pressed down to mold it accurately to the scalp. The printed positive mold of the scalp is shown in Fig. 12 on the right and the molded Aquaplast RT is shown on the left.

## IV. CONCLUSIONS

PDD measurements and CT data concluded that the correct Hounsfield unit to use for our clear‐PLA printing material is 260. This was further confirmed by the dose plane comparison which models a real treatment. Considering these results, we are confident that we can accurately model this printing material in our treatment planning system for all energies in photon and electron beams. The 3D printer has also been useful in other ways. In the event the patient cannot have bolus materials placed on their skin for molding, we can print a 3D positive mold of the patient's treatment area, molding the bolus to the replica instead. This process of printing our own bolus streamlines patient care, minimizes patient involvement, and maintains quality treatments.
